# PHYSICAL ACTIVITY AND EXERCISE-INDUCED NEUROTROPHIC AND CORTISOL RESPONSES: PRELIMINARY RESULTS

**DOI:** 10.1093/geroni/igae098.3490

**Published:** 2024-12-31

**Authors:** Thinh Huynh, Baylie Rushing, Madeleine Love, Denise Head, Marta Stojanovic

**Affiliations:** Washington University in St. Louis, St. Louis, Missouri, United States; Washington University in St. Louis, St. Louis, Missouri, United States; Washington University in St. Louis, St. Louis, Missouri, United States; Washington University in St. Louis, St. Louis, Missouri, United States; Washington University in St. Louis, St. Louis, Missouri, United States

## Abstract

Neurotrophic and cortisol responses to a bout of exercise are a pathway for understanding the potential mechanisms underlying the beneficial effects of regular physical activity on cognitive and brain health. However, there is limited understanding of the factors that moderate such responses. This study examined whether long-term exercise history and current moderate-to-vigorous physical activity (MVPA) levels predicted neurotrophic and cortisol responses to acute exercise. In addition, we investigated whether these associations were moderated by age and sex. Clinically normal middle-aged and older adults from the Knight ADRC Adult Children Study (n=54; age=43-85) completed a questionnaire about exercise engagement over a 10-year period and underwent a submaximal VO2 assessment, with both representing a combined estimate of their long-term physical activity. Participants also completed seven days of actigraphy, which was used to estimate current MVPA. Participants engaged in a bout of exercise, with neurotrophic factors and cortisol measured at baseline, 10 minutes post-exercise, and 30 minutes post-exercise. Long-term physical activity did not influence neurotrophic (p=0.538) or cortisol responses (p=0.276), regardless of age and sex (p’s >0.143). Higher current MVPA was associated with a greater neurotrophic response (p=0.007) across sex (p=0.165). Additionally, there was a moderation by age (p=0.014), with less of an association as age increased. Greater current MVPA was also associated with a smaller cortisol response (p=0.044), across age and sex (p’s>0.144). These results suggest that current MVPA may be particularly important when considering pathways between a single bout of exercise and neurocognitive health.

